# Polymicrogyria: pathology, fetal origins and mechanisms

**DOI:** 10.1186/s40478-014-0080-3

**Published:** 2014-07-22

**Authors:** Waney Squier, Anna Jansen

**Affiliations:** Department of Neuropathology, Level One, West Wing, Oxford University John Radcliffe Hospital, Oxford, OX3 9DU UK; Paediatric Neurology Unit, Department of Paediatrics, UZ Brussel, Brussels, Belgium; Neurogenetics Research Unit, Vrije Universiteit Brussel, Brussels, Belgium

**Keywords:** Polymicrogyria, Cortical malformation, Fetal brain development, Neuropathology, Leptomeninges

## Abstract

Polymicrogyria (PMG) is a complex cortical malformation which has so far defied any mechanistic or genetic explanation. Adopting a broad definition of an abnormally folded or festooned cerebral cortical neuronal ribbon, this review addresses the literature on PMG and the mechanisms of its development, as derived from the neuropathological study of many cases of human PMG, a large proportion in fetal life. This reveals the several processes which appear to be involved in the early stages of formation of polymicrogyric cortex. The most consistent feature of developing PMG is disruption of the brain surface with pial defects, over-migration of cells, thickening and reduplication of the pial collagen layers and increased leptomeningeal vascularity. Evidence from animal models is consistent with our observations and supports the notion that disturbance in the formation of the leptomeninges or loss of their normal signalling functions are potent contributors to cortical malformation. Other mechanisms which may lead to PMG include premature folding of the neuronal band, abnormal fusion of adjacent gyri and laminar necrosis of the developing cortex. The observation of PMG in association with other and better understood forms of brain malformation, such as cobblestone cortex, suggests mechanistic pathways for some forms of PMG. The role of altered physical properties of the thickened leptomeninges in exerting mechanical constraints on the developing cortex is also considered.

**Electronic supplementary material:**

The online version of this article (doi:10.1186/s40478-014-0080-3) contains supplementary material, which is available to authorized users.

## Introduction

The name polymicrogyria (PMG) implies cortical gyri which are too many and too small, but the pathology, imaging and clinical manifestations of PMG are heterogeneous [1]. PMG may be focal or widespread; the extent and distribution of the abnormality will determine its clinical manifestations. Clinical, radiological and pathological studies stress the phenotypic heterogeneity [2,3]. PMG is similarly aetiologically heterogeneous, resulting from both genetic and destructive events, including infection, hypoxia-ischaemia, and trauma [4] and cases with similar morphology do not necessarily share aetiologies. Genetic mutations have been identified in association with PMG, but in none is PMG the sole or specific abnormality. A single PMG syndrome may have multiple genetic aetiologies and, conversely, single genetic causes may give rise to variable PMG patterns [5-7]. Even combined analysis of clinical and imaging features, pathology and genetics fails to identify the underlying aetiology in the majority of cases. These features strongly suggest that PMG is not a single entity but instead represents the common endpoint of a variety of aberrations of cortical development.

In order to understand how and when such a heterogeneous condition occurs the specific developmental pathways which are disrupted need to be identified. The time of onset of PMG has not been established; it has been suggested to be both early, due to impaired proliferation and migration of neuroblasts [8,9] and late, due to disordered post-migrational maturation of the cortex [10]. The vast majority of studies of PMG and its clinical diagnosis have been based on radiological studies of the mature brain. Examining the post-natal brain, long after development is complete, is unlikely to reveal pathogenetic mechanisms; acute reactions have by then subsided and maturation and myelination obliterate earlier pathology [1,11]. Animal models cannot be directly extrapolated to the developing human brain. Studies of fetal human brains are uniquely able to demonstrate human cortical development and its aberrations while they are in progress. Few such studies have so far been undertaken.

This review is based on extensive examination of human neuropathology, predominantly of fetal brains. We discuss the mechanisms which may lead to these patterns of abnormal cortical folding and describe the patterns of pathology seen in PMG of known aetiology (Additional file 1).

### Pathological definition of PMG

**Figure 1:**
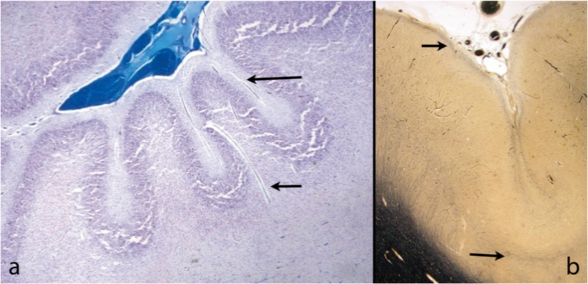
**Typical features of PMG in mature brains. a**: Term baby; a festooned band of nerve cells beneath a slightly bumpy surface with normal leptomeninges. Several large intracortical vessels are seen (arrows). Little myelin is seen at this age. (Luxol fast blue and cresyl violet (LBCV)). **b**: 16 year old. The myelin stain (Loyez) shows a band of myelinated fibres on the cortical surface indicating disruption of the anatomy at this level as well as in the neuronal band (arrows). The leptomeninges are adherent but not markedly thickened. The deep cortical junction with the white matter is sharp.

Friede defined PMG as *“an abnormally thick cortex formed by the piling upon each other of many small gyri with a fused surface”*. The characteristic microscopic features he described were abnormal arrangement of cells, an intracortical fibre plexus, excessive folding of all or only the upper layers, and fusion of gyral surfaces with large sinusoidal intracortical vessels [12] (Figure 1) Norman [13] in a more detailed description, identified five subtypes: 1 - unlayered ‘festooned’, 2 - four-layered cortex with a sinuous upper layer, 3 - parallel four-layered cortex, 4 - miniature gyri which are fused and 5 - poorly laminated. Fusion was only specified in “miniature gyri” but could be seen in combination with other patterns. Judkins [14] proposed that the minimal criteria for PMG should be fusion of the molecular layer with *“festooning of the cortical surface, independent of other cortical changes*”.

Cortical layering has been further classified as two, four and unlayered forms which are thought to reflect the time of insult. Unlayered is said to be “early”, occurring at 16–24 weeks, and 4-layered is late [15]. In early fetal cases it can be very difficult or impossible to define the number of layers in the cortex. In older cases, multiple layering patterns are commonly encountered in the same case and there is considerable overlap between them. Judkins considered that classification of PMG according to cortical lamination was unhelpful [14].

It is clear that both fusion and cortical lamination are highly variable between cases and may not be reliable criteria for definition of the malformation. Some authors do not use the term PMG in the absence of fusion [16] while others do [17]. Our approach in reviewing this subject has been inclusive and we have accepted as PMG any case with festooning of one or more cortical layers.

PMG can occur as an isolated finding but almost always it is associated with other anomalies of the central nervous system (CNS) including periventricular nodular heterotopia, excessive scattered neurons in the underlying white matter, agenesis of the corpus callosum and brainstem or cerebellar abnormalities. It can also be part of a multiple congenital anomaly syndrome with additional abnormalities outside the CNS.

### Radiological definition of PMG

**Figure 2:**
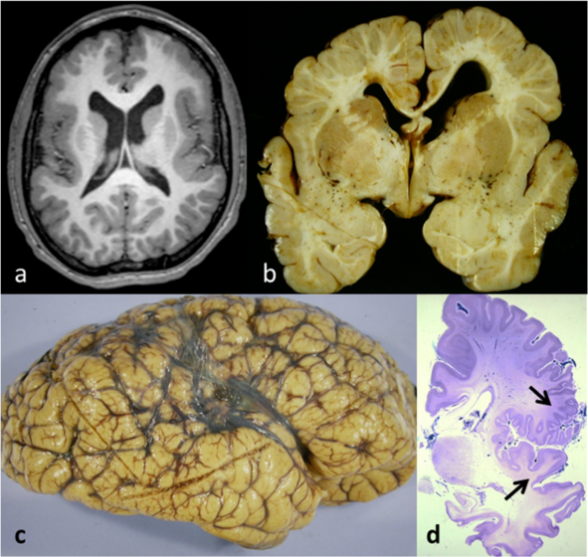
**PMG macroscopic appearance in MRI and fixed brain. a**: MRI; horizontal image. The cortex over the insular and frontal areas is polymicrogyric; irregular with many small, densely-packed folds, while the cortex of the posterior brain (below) is normal. **b**: Coronal slice through a fixed brain shows small, densely-packed cortical folds over the frontal lobes but the cortex of the basal temporal lobes is spared. The corpus callosum is thin and the ventricles are large. **c**: Lateral view of a fixed brain; the gyri are coarsely nodular. The naked eye appearance of polymicrogyria can be subtle and requires histological confirmation. **d**. Section of hemisphere with abnormally festooned cortex with multiple small folds in the Sylvian fissure (between black arrows) (LBCV stain).

Based on imaging, PMG has been classified into three morphological subtypes: coarse, delicate and saw-toothed, although the appearances are highly variable and depend on age and degree of myelination [1,3] (Figure 2).

Radiological delineation of patterns of PMG is valuable in the clinical evaluation of patients and directing genetic studies, but in general the radiological appearances correlate poorly with aetiology. Radiology does not have the resolution to describe the detail of the malformed cortex; pathological correlation is essential for its accurate interpretation. This is not often done, as many cases survive into adult life and autopsies are rarely performed in them; material derived from epilepsy surgery is restricted to patients whose epilepsy was so severe as to necessitate surgery. In either case only the most severe or long-standing cases, likely to be complicated by secondary atrophy and gliosis, are studied and the pathology of milder phenotypes remains unclear. In a recent reclassification of the radiology of PMG, Barkovich [1] called for detailed description of the pathology of PMG and we attempt to address this.

### Mechanisms of PMG

Taking a broadly inclusive approach, and accepting as PMG any case where one or more cortical layers shows abnormal folding or festooning, the most common pathology is of disruption of the cortical surface and its interaction with the leptomeninges. Other patterns include fusion of the molecular layers of adjacent gyri, premature folding of the cortical neuronal band and early destructive lesions.

#### Disorders of the brain surface

The brain surface is complex, consisting of the leptomeninges (arachnoid membrane, pial cells), basement membrane, radial glial/astrocytic end feet and Cajal-Retzius cells in the upper molecular layer. All of these elements have been implicated in cortical malformations.

The fetal meninges play an important role in cortical development; they regulate cortical neuronogenesis, cell migration and positioning, and maintain the pial basement membrane, which is critical for attachment of radial glial end feet [18]. Very early animal experiments demonstrated that compromising the integrity of the brain surface led to cortical malformation, including PMG. Freezing the upper cortical layers or gentle brushing of the uncovered meninges in newborn rats caused PMG, while the same insults after day 4 of life caused necrosis [19,20]. Surgical removal of the meninges and their associated blood vessels in fetal mice led to apoptosis of radial glial cells and reduction in cortical size [21].

**Figure 3:**
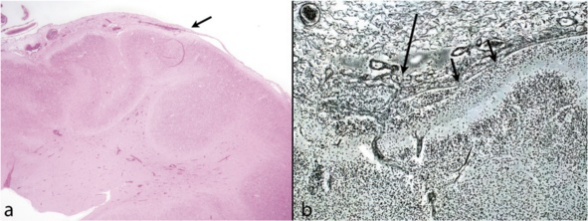
**Leptomeningeal changes over PMG cortex. a**: The leptomeninges become adherent, thickened and vascular (arrow) as the cortex beneath them becomes abnormally festooned towards the left of the image. Mature brain (Haematoxylin and Eosin (H&E)). **b**: Fetus of 19 weeks: high power image shows reticulin stain to show the thickened leptomeninges over PMG cortex. The pial basement membrane is adherent and shows focal gaps with cells entering the leptomeninges from the subpial layer (short arrows). A vessel is seen crossing the pia between hypercellular leptomeninges and the cortex (long arrow) (reticulin).

Abnormal leptomeninges are seen overlying human PMG [22], and leptomeningeal thickening with vascular proliferation, pial defects and over-migration, are seen in over 80% of cases of PMG, both of genetic and destructive origin [Jansen A, Robitaille Y, Honavar M, Mullatti N, Leventer RJ, Andermann E, Andermann F, Squier W (forthcoming) The histopathology of polymicrogyria: a series of 71 brain autopsy studies] (Figure 3). It remains to be clarified whether leptomeningeal pathology participates in the genesis of PMG or represents a secondary change. Acute infarction per se causes reactive change in the leptomeninges which may cause fusion of adjacent gyral surfaces without cortical malformation. The determining factor appears to be the timing of the insult [19].

Adhesion of the medial frontal lobes in PMG [23,24] implies not only abnormal surface fusion, but also failure of the normal formation of the midline falx and primitive venous sinuses which are already present between the medial aspects of the frontal lobes by six weeks of gestation [25]. This suggests that all three meningeal layers, not just pia/arachnoid may be involved in normal corticogenesis.

### The pial basement membrane

For over a decade it has been recognised that an intact pial basement is required for proper cortical histogenesis [26]. An increasing number of cortical dysplasias, including PMG, have been associated with defects in specific basement membrane components, these include fibronectin, laminin γ-1, laminin α2 and α4, Col4A1 and α-dystroglycan [21,26-29]. Animal models indicate that the basement membrane appears to develop normally at first but later defects appear as a result of instability during brain growth and remodelling [29-31].

### Pial cells

Pial meningeal cells are derived from the neural crest and migrate in a ventral to dorsal direction over the developing mammalian forebrain. They produce a number of signalling factors (including CXCL12, Zic and retinoic acid) which play an important role in neuronal migration and positioning in the cortex and in the development of the astrocytic end feet of the superficial glia limitans. Pial cells also secrete components of both the interstitial matrix and the basement membrane [18,32]. *FOXC1* is expressed in all three meningeal layers, and in humans is associated with defects in eye and cerebellar development [33]. A mouse model of Foxc1 mutation demonstrates a direct relationship between impaired migration of meningeal cells and cortical malformation; areas of cortex covered by leptomeningeal cells develop normally but areas of the cortex where leptomeningeal cells fail to migrate are malformed [34]. In this model the most severe phenotype showed widespread cortical disruption but the basal cortex was spared. In the less severe phenotypes the malformed cortex was only found over the dorso-lateral cerebral cortex, corresponding to areas where meningeal cells had failed to migrate. The resulting pattern of cortical malformation mimics the typical distribution of PMG in human cases, with preferential involvement of the dorsolateral cortex, sparing the basal cortex [3,17]. Perturbation of signals produced by meningeal cells also causes disruption of tangential migration and laminar and regional distribution of cortical interneurons, and interferes with development of the corpus callosum [35-37].

### Cajal-Retzius (CR) cells

Cajal-Retzius (CR) cells have a role in normal corticogenesis [38] and may be focally increased in number, persisting beyond fetal life, in areas of PMG [39]. CR cells originate medially in the cortical hem and migrate over the cerebral hemispheres, the most distal cortical fields being the lateral hemisphere (future perisylvian region) [40]. The meninges are necessary as a physical substrate for CR cell migration and also as a source of the chemoattractant CXCL12 [41]. The frequent occurrence of PMG in the distal parts of this pathway is not explained simply by failure of CR migration as animal models have identified CR cells in the abnormal cortical regions. However, the CR cells are displaced into the leptomeninges, where it is possible that their function may be impaired [29,31,34].

### Radial glial cells/astrocytic end feet

The importance of the radial glial cells (radial precursor cells) for neuronal migration and normal and abnormal cortical formation is well established [42]. The integrity of the attachment between radial precursor end feet and the brain surface appears to be essential for their survival and, if damaged, leads to apoptosis and reduction in cortical size [21]. PMG is commonly associated with abnormalities of neuronal migration including heterotopia [1,43,44].

#### Fusion of the adjacent gyral surfaces

The traditional explanation for PMG has been that it comes about by fusion of adjacent cortical gyri. Indeed, Judkins proposed that the minimal criteria for PMG should be fusion of the molecular layer with festooning of the cortical surface, independent of other cortical changes [14].

**Figure 4:**
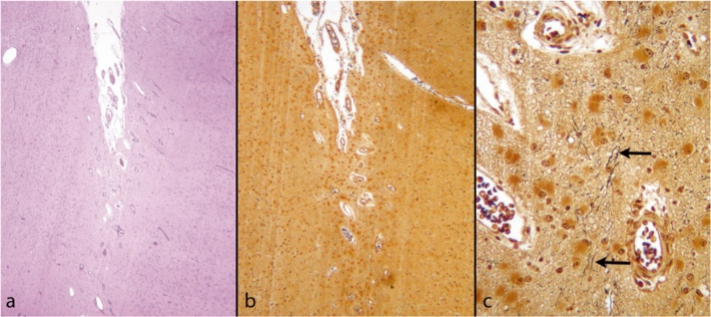
**Cortical fusion; fetus 34 weeks.** Depths of a sulcus at low power (**a**. H&E stain, **b**. Reticulin) shows the sulcus extending into the cortex as a seam. There are abnormally large blood vessels in the superficial cortex around the seam. **c**: At higher power the reticulin stain shows collagen fibres (arrows) which are not found in normal cortex and represent the remnants of trapped leptomeninges. There are abnormal large blood vessels and many plump, reactive astrocytes.

Importantly, there is a question of how fusion is defined. Fusion is frequently described in the various forms of PMG, but Norman specified it in only one of the five subtypes she described [13]. Friede’s definition of PMG included *“large sinusoidal vessels marking the seams between adjacent fused gyri”* [45]. However, large vessels may merely be a part of a field malformation and cannot in themselves be an indication of fusion; they are identified in cobblestone cortex where fusion is not a mechanism [11,46,47]. We suggest that fusion may be defined only when there is additional evidence of trapped remnants of the leptomeninges between the folds of the festooned neuronal band. We have based our definition of true fusion on the observation of bundles of collagen fibres, which are normal in the pial basement membrane and around blood vessels, but are not normal in the brain parenchyma (Figure 4).

While the vestiges of gyral fusion are commonly seen, fusion is by no means universal in PMG [17]. In one form of PMG, associated with periventricular heterotopia, multiple sulci appeared to demonstrate different stages of fusion with adjacent gyri “zipping up” from the depths of sulci. The leptomeninges were normal but increased numbers of cells were identified in the subpial zones at the bases of “zipping” adjacent sulci [48].

#### Premature cortical folding

Little is known of the signals for, or mechanisms of, normal cortical folding. Many hypotheses have been proposed during the last century, but no clear mechanism is agreed.

Studies of the fetal cortex need to take into account the normal appearances at different gestational ages. The fetal brain is normally smooth until normal gyration begins at 21 weeks gestation. Gyration is well defined by 28 weeks and by term is almost complete. It can be impossible to identify abnormal folding patterns, particularly by radiology, before this time. Abnormal undulation of the upper cortical layers between 18 and 28 weeks of gestation may be mistaken for PMG but this finding (Papillae of Retzius or status verrucosus), is most probably an artefact.

**Figure 5:**
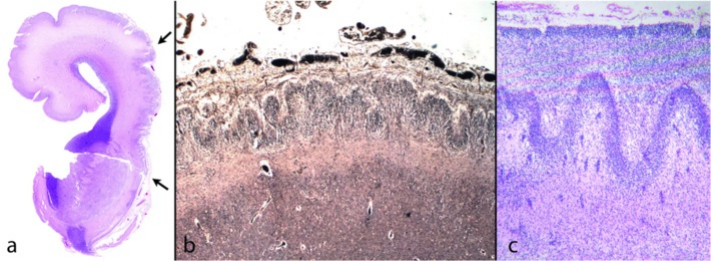
**Premature cortical folding. a**: Fetus of 18 weeks with an area of abnormal cortical folding in what will become the perisylvian area (arrows). The surface of the brain elsewhere is smooth, as expected at this age (H&E). **b** and **c**: two examples of abnormally festooned neuronal bands in fetuses aged 29 and 26 weeks. In **(b)** (29 weeks) a single neuronal band festoons below thickened and very vascular leptomeninges (reticulin). In **(c)** (26 weeks) a single neuronal band festoons below a straight subpial, densely cellular, band. Less densely packed cells occupy the tissue between these two more cellular bands.

Not uncommonly abnormal festooning of the cortical neuronal band occurs well before the time at which normal cortical folding is expected (Figure 5).

**Figure 6:**
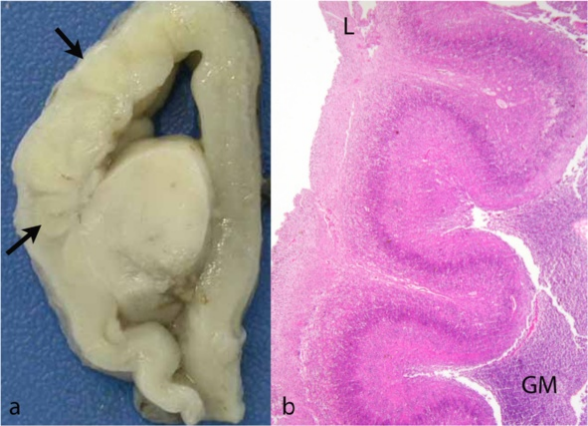
**Undulating band heterotopia; fetus of 23 weeks. a**: A slice of the fixed brain shows large ventricle and a thin cortical mantle in which the undulating cortical neuronal band can just be made out (arrows). **b**: H&E stained section shows a festooned band of neurones sweeping from the brain surface (L) to the ventricular zone where germinal matrix (GM) separates it from the ventricular wall.

In a more extreme example the entire developing cortical wall is occupied by a festooned band of neurones, organised into three layers, sweeping between the brain surface and the germinal matrix lining the lateral ventricle. Fusion of adjacent folds is not seen and the cortical surface is normal (Figure 6). This appearance has been labelled undulating band heterotopia [49]. In one study loss of doublecortin was identified in the neurones of the subcortical band [50].

#### Temporospatial patterning of PMG

Radiological studies have elucidated specific patterns of distribution of the PMG [3,1]. It occurs most commonly in the perisylvian region; the dorsolateral or frontal regions are also frequently involved but the base of the brain and the medial temporal cortex and the hindbrain tend to be spared. On the contrary, the pathological literature has paid little attention to the spatial distribution of PMG.

The potential role for migration of leptomeninges and CR cells in the patterning of PMG has been discussed. The spatial patterning of the cortex is under genetic control and one transcription factor responsible for the cortical area map, EOMES (eomesodermin), has been shown to be defective in a syndrome which includes PMG, microcephaly and agenesis of the corpus callosum [51,52].

Other, better studied, cortical malformations may throw light on the mechanisms and spatial distribution of PMG. In recent years the distinction between cobblestone malformation and PMG has become blurred and focal PMG is frequently seen together with cobblestone lissencephaly [1,24,53,46,47]. The cobblestone malformation is the prototype of cortical overmigration into the leptomeninges in association with defects in the pial surface of the brain [11]. Originally described in association with congenital muscular dystrophy and dystroglycanopathy, pial basement membrane defects and over-migration are now recognised in association with mutations in numerous genes including *GPR56, TUBB2B, COL4A1* and *MARCKS* [7,26,28,29,31,54]. Disruption of the pial basement membrane is identified in the majority of cases of PMG of all causes [Jansen A, Robitaille Y, Honavar M, Mullatti N, Leventer RJ, Andermann E, Andermann F, Squier W (forthcoming) The histopathology of polymicrogyria: a series of 71 brain autopsy studies].

**Figure 7:**
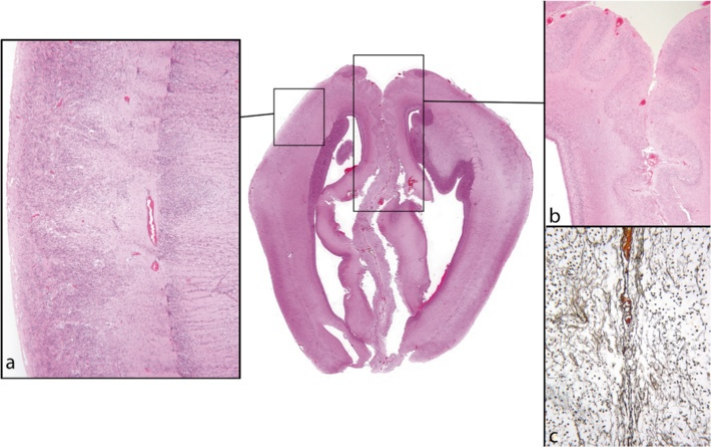
**Cobblestone malformation; fetus aged 20 weeks.** In the centre is a coronal section through the frontal part of the brain. The classic cobblestone cortex is seen over the lateral hemisphere walls (box **a**). There is a gradual transition in the nature of the malformation over the vertex and into the medial frontal lobes where the appearance is of PMG. The adjacent medial frontal surfaces are fused and share a basement membrane (boxes **b** and **c**) (H&E). **a**: Higher power of classic cobblestone malformation in the lateral wall of the frontal lobe. A line, representing the original pial surface, runs vertically through the mid region of the cerebral wall. To the left the lateral brain surface consists of masses of ectopic neurones mixed with vascular meninges. To the right in the deeper part of the brain wall clusters of neurones of the original cortex directly abut the pial surface (H&E). **b**: Medial frontal lobes. There is no falx. The cortical neuronal band is festooned. The leptomeninges are thickened and dip into fused adjacent folds of the neuronal band. **c**: The surface collagen layers are reduplicated and fused in the midline (reticulin).

Cobblestone lissencephaly, the malformation in *GPR56* and *TUBA1A* mutations as well as PMG tend to spare the base of the brain and inferior temporal regions. In cobblestone lissencephaly the most severe cobblestone pathology is over the lateral aspects of the hemispheres, but the pattern undergoes a subtle transition in the dorsomedial region to a different pattern in the medial frontal lobes where PMG and surface adhesion may be seen [16,23,24,53]. The adjacent medial frontal surfaces are often fused or interdigitated with microscopic bridges of connective tissue containing immature cells extending between them (Figure 7). The spatial distribution of the cortical malformation is replicated in the *Foxc1* mouse model [18]. Fusion of the medial frontal lobes further indicates failure of development of the midline meningeal falx, emphasising the importance of all layers of the meninges for normal cortical development. Interestingly, patients with deletions of 6p25.3, which codes for FOXC1, show defects of the midline dural folds (falx and tentorium) [33].

An obvious difference between PMG and the cobblestone malformation is sparing of the cerebellum and brainstem in the vast majority of PMG. Unlike classical lissencephaly, in the cobblestone malformation the brainstem and cerebellar cortex, which depend on migration pathways through the leptomeninges, are malformed [55]. There are at least two potential explanations for this. The leptomeninges in these areas have separate origins; the forebrain meninges are derived from the cranial neural crest, while the hindbrain and spinal cord leptomeninges arise in the mesoderm [56]. Secondly, neuronal migration in the forebrain is largely dependent on radial precursor cell guides, while in the hindbrain a large proportion of migration is dependent on adhesion to the surface basement membrane [27,57,58].

The temporal effects of migration and specific gene expression may be equally important; PMG develops after insults in specific developmental periods [19]. Similarly, the death of radial glial cells is triggered by removal of the meninges in animal models only in very specific time frames [21]. *GPR56* mutation causes both PMG and a cobblestone like pathology [31] and also involves the cerebellum. In *GPR56* null mice the rostral cerebellar defect appears to depend on specific failure of adhesion of the late migrating cortical granule cells to the basement membrane, indicating developmentally critical differences between regions, some existing only transiently [27]. It is notable that posterior cerebellar agenesis or hypoplasia is also a feature in *TUBA1A* mutation [16].

### Physical and mechanical implications of surface pathology

A further mechanistic consideration is the mechanical constraint that abnormal leptomeninges may place on the developing cortex. One of the commonest pathologies in PMG is thickening of the leptomeninges overlying the malformed cortex. This may impose physical constraints on the expanding cortex, forcing the developing neuronal band to undergo increased folding in order to be accommodated in an area where expansion is limited by the rigidity of the overlying leptomeninges. (Personal communication, Alain Goriely OCCAM, Oxford).

### PMG and associated aetiologies

#### PMG associated with congenital infections

**Figure 8:**
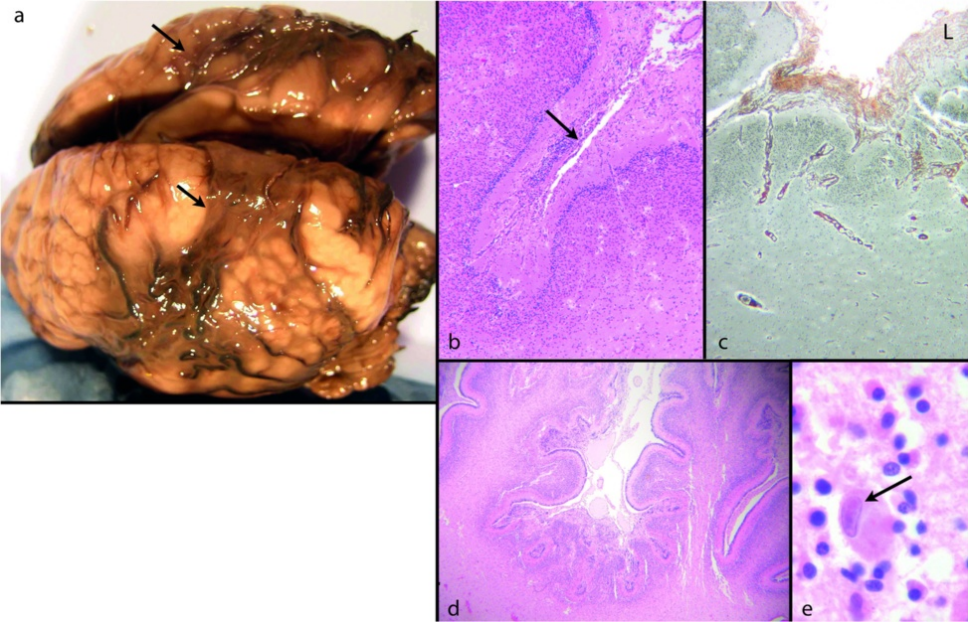
**CMV; fetus 35 weeks. a**: Fixed brain showing a well-defined area of cortical malformation in the peri-Sylvian region (arrows). **b**: The cerebral cortex shows increased cellularity in the molecular layer of the cortex (arrow) (H&E). **c**: Reticulin stain shows the increased collagen in the leptomeninges and around blood vessels dipping into the cortex. **d**: The cerebellar cortex is also involved and abnormally folded with extensive disruption of its superficial layers (H&E). **e**: A typical large nuclear inclusion of CMV (H&E).

**Figure 9:**
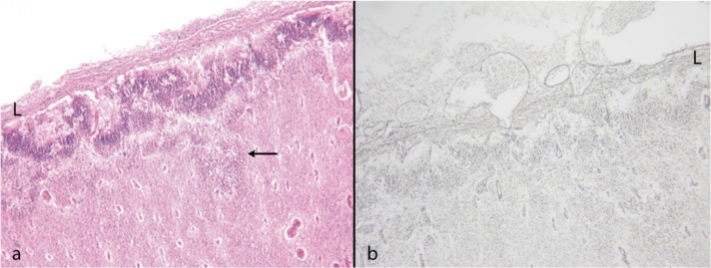
**CMV; fetus 22 weeks. a**: The developing cortical plate is an undulating band beneath thickened leptomeninges (L). A mass of incompletely migrated cells merges into the irregular deep cortical border (arrow) (H&E). **b**: Reticulin stain shows dilated surface vessels and multiple layers of reticulin overlying an irregularly festooned neuronal band.

Cytomegalovirus (CMV) is the commonest congenital infection and a common infectious cause of PMG [12,59]. Consistent microscopic features include prominent pathology at the brain surface overlying the polymicrogyric cortex (Figure 8). Necrosis, calcification and meningeal inflammation associated with damage to the glia limitans are described [60]. We have observed reduplicated layers of pial reticulin with focal defects and overmigration of cells as well as increased cellularity and dilatation of blood vessels of the leptomeninges. The deep cortical border is irregular, sometimes with nodules or columns of incompletely migrated cells in the underlying white matter (Figure 9). In older cases trapped, thickened leptomeninges, basement membrane and blood vessels within a festooned cortical neuronal band suggest previous fusion of adjacent gyral surfaces.

The characteristic features of CMV (giant nuclear inclusions, inflammation, microglial clusters and calcification) are usually easily identified. In establishing an infectious cause the distribution of PMG may be helpful. While the distribution of pathology over the cerebral hemisphere is not consistent, both the cerebral and the cerebellar cortex are usually involved, as well as the eyes and other organs. The pathology in the 35 week fetal brain shown in Figure 8, with preferential damage in the perisylvian cortex, was initially interpreted as due to a genetic or vascular cause [4]. The cerebellar cortex was also severely damaged.

Failure to identify the hallmarks of infection does not indicate that infection can be ruled out. One of our cases was a baby born at 35 weeks and dying at 8 weeks of age from cardiorespiratory failure with small, well-developed foci of PMG with a festooned neuronal band. There was sparse calcification and a single microglial cluster was identified. Despite a careful search, including with immunohistochemistry, no cells typical of CMV were identified in the brain. It was only on subsequent histology of the body organs that CMV in the kidney and pituitary gland was identified. CMV should still be considered as the aetiological agent even when it cannot be identified in the brain and may be a more common cause of PMG than is currently recognised.

A further complication in establishing a diagnosis of CMV is that this infection is capable of recurring in subsequent pregnancies, which might be taken to suggest a genetic cause. In one study the rate of transmission to infants born to mothers who had a primary infection or a recurrent infection during pregnancy was 32% and 1.4%, respectively [61]. While the manifestations in the infant are generally considered to be more severe in primary infections, fetal death and severe neonatal illness have been described following congenital infection in seropositive mothers [59].

Teissier et al. [60] have shown that CMV infects all cell types in the brain but shows higher tropism for stem cells/radial glial cells. This suggests that the large spectrum of CMV-induced brain abnormalities is caused not only by tissue destruction but also by the particular vulnerability of stem cells and radial glia during early brain development. This is in line with earlier studies showing damage to vascular endothelium [62] and with mouse models which have shown that neural and progenitor cells in the subventricular zone are the most susceptible to CMV. In later stages the infection may involve neurones and become latent [63].

Toxoplasmosis has a less commonly described, but recognised, association with PMG [45].

### PMG associated with ischaemia

**Figure 10:**
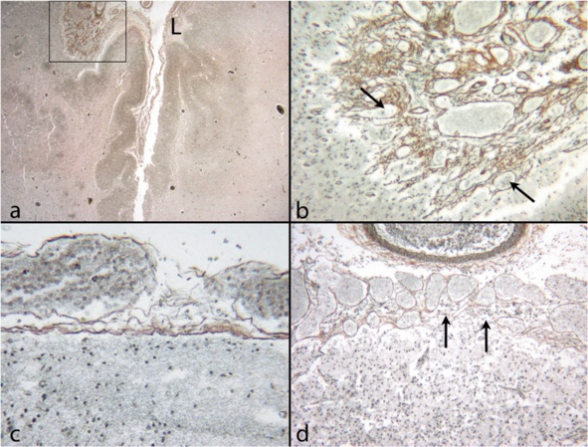
**Ischaemia; fetus of 36 weeks following trauma at 24 weeks gestation. a**: A cleft, bordered with irregularly festooned cortex, dips into the brain from the surface. The cleft is immediately adjacent to the infarcted middle cerebral artery territory (box) where there is thickening and increased vascularity of the overlying leptomeninges (reticulin). **b**: Higher power of the pial surface from area within box in **(a)** showing apparent loss of pial basement membrane, multiple layers of collagen and large blood vessels. Abnormal masses of cells (presumed over-migrated) are seen between these vessels (arrows). **c**: An area of normal cortex from the same case stained with reticulin to show the pial basement membrane and surface blood vessels. **d**: Reactive surface overlying infarcted cortex. The pial basement membrane no longer forms a discrete band but is irregular and thickened with focal breaches (arrows). The surface blood vessels are numerous and dilated.

PMG associated with ischaemic damage is usually identified on the border of areas of infarction but may also occur either within arterial territories or quite distant from areas of frank infarction [9,22,64]. Rarely in human cases is there precise timing of an ischaemic event leading to cortical malformation. In a case reported in detail elsewhere [48], a mother suffered a road traffic accident at 24 weeks gestation and pregnancy was terminated 12 weeks later because fetal MRI showed development of bilateral “porencephalic cysts” and subdural haemorrhages. The baby’s development was otherwise normal; there was no evidence of direct trauma to the baby, placental infarction or haemorrhage. Pathology confirmed bilateral middle cerebral artery infarction. Histology showed PMG with pial basement membrane reduplication, over-migration into the leptomeninges and increased leptomeningeal vascularity bordering the infarction and in the occipital watershed zones. There was also subcortical heterotopia, transmantle dysplasia, and schizencephaly (Figure 10). The brain damage was the result of fetal cerebral ischaemia, rather than direct trauma to the baby or the brain. This case illustrates that PMG may occur after a single ischaemic insult at 24 weeks and be associated with leptomeningeal disruption and over-migration as well as a complex spectrum of cortical malformations.

#### Watershed zone PMG

PMG associated with ischaemia has classically been described in arterial territories, but is rarely described in a watershed distribution [65,66]. This indicates that watershed perfusion failure may occur before the twenty-fourth week of gestation, when PMG is considered to develop, and is not restricted to the term brain as often suggested [67].

#### Laminar necrosis

Ischaemic aetiology is usually assumed when necrosis is seen in a distribution which conforms to a known vascular territory or when a mutation likely to impair vascular function is identified (see below). Due to immaturity of the vascular system, destructive lesions of the fetal brain may be less clearly anatomically defined than in later life. When other causes can be excluded, e.g. infectious agents are not identified by histological or microbiological means; destructive lesions are generally assumed to be ischaemic in origin. Infarction or haemorrhage in other organs or the placenta supports a systemic disease such as coagulopathy, vasculopathy or placental pathology.

**Figure 11:**
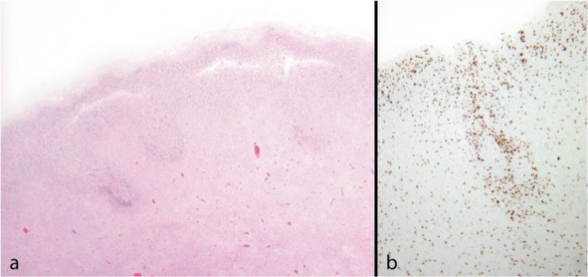
**Laminar necrosis; fetus 22 weeks. a**: There is a horizontal split in the cortex. The upper cortical layer above the split is mildly irregular, the lower layer has an ill-defined deep border with clusters of incompletely migrated cells merging into it. **b**: CD68 stain shows large numbers of macrophages in the cortex and the subcortical masses of cells.

Necrosis of specifically vulnerable cortical layers has been suggested as a cause of two and four-layered PMG [8,15,68]. In fetal laminar necrosis we have observed a horizontal split separating the cortex into two bands (Figure 11). Either may be festooned; the deep band is often irregular and in continuity with groups of incompletely migrated cells extending into the underlying white matter. Laminar necrosis is associated with thickening, fibrosis and increased vascularity of the arachnoid [68]. There may also be reduplication of the pial basement membrane. Increased numbers of macrophages, some containing haemosiderin or intracellular calcification, may be identified in the cortical split as well as among the clusters of cells in the white matter. These support the diagnosis of a destructive aetiology.

The reason for irregular folding of the residual layers is unclear; Dvorak [19] proposed that cells migrating through damaged tissue are misplaced in the mature cortex. The large numbers of macrophages may be significant; they have been shown to play a part in axonal guidance and synaptogenesis [69].

#### Arterial calcification

**Figure 12:**
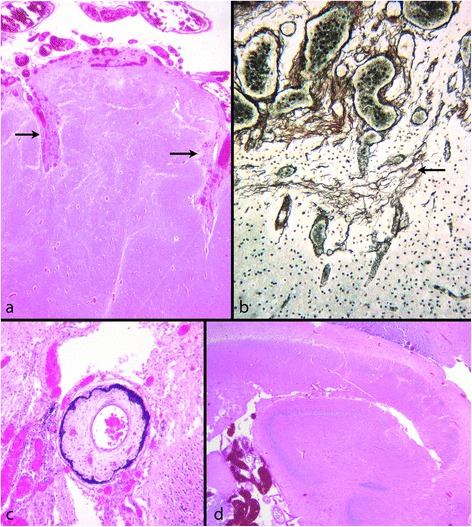
**Arterial calcification; fetus 36 weeks. a**: H&E stain showing festooned cortex with adhesion of the leptomeninges to the cortical surface The thickened, vascular meninges dip into the sulci (arrows). **b**: Reticulin stain showing thickened pial collagen and increased leptomeningeal vascularity. The pial surface is partly buried in masses of heterotopic cortex (arrow). **c**: Surface artery with confluent calcification in the wall. The intima is thickened and fibrous with a very reduced lumen. **d**: The hippocampal cortex is normal.

We have examined one 35 week male fetus with extensive PMG associated with a very unusual vascular anomaly (Figure 12). Large and medium sized meningeal arteries had adventitial calcification with intimal thickening and significant luminal narrowing. The baby was microcephalic, with extensive PMG over the cerebral hemispheres. There was pial BM reduplication, over-migration and increased leptomeningeal vascularity. The hippocampus was preserved and the cerebellar cortex normal but there was dentato-olivary dysplasia. There was patchy tissue calcification in the cerebral cortex, white matter and deep grey matter typical of old necrosis, but no evidence of intrauterine infection. No gene testing was undertaken. The vascular appearances are very occasionally seen in leptomeningeal vessels of babies with prolonged survival after hypoxic-ischaemic brain damage. They are also described in the systemic vessels in the syndrome of “Generalized arterial calcification of infancy” in mutations in either *ENPP1* or *ABCC6* [70]. Involvement of cerebral arteries in this syndrome appears to be most unusual; of 180 cases so far published one female infant of 37 weeks gestational age was shown by ultrasound examination to have calcification in the cerebral vessels [71]. No case has yet been described with PMG. Reduced cerebral vascular compliance due to arterial calcification leading to ischaemia at the appropriate developmental stage may provide an explanation for the cortical dysplasia in this unusual example.

#### Schizencephaly

Schizencephaly is a form of destructive brain lesion which may be seen in association with PMG. It is defined as a cleft of the brain wall extending from the brain surface to the ventricular lining [72] but is not clearly distinguished from porencephaly or other destructive lesions in the developing brain. The term carries no aetiological implications.

### Ischaemia of multifactorial or genetic origin

Ischaemia of the developing brain is usually acquired but may also be multifactorial or genetic in origin. Several genetically determined conditions are associated with PMG of ischaemic origin.

#### Occludin

PMG has been identified in patients with mutations in the occludin gene *(OCLN)* in a syndrome which also includes band-like calcification and simplified gyration. Occludin is an integral component of the tight junctions of endothelial cells in the brain. It is also involved in organization and orientation of the microtubular network in epithelial cell migration during wound healing [73], but does not appear to have been implicated in neuronal migration.

MRI of patients with occludin mutations shows characteristic bilateral, symmetrical, predominantly fronto-parietal PMG, a prominent band of calcification in the cortex, and calcification in the cerebellum and basal ganglia. Neuropathology in one case has shown the calcification predominantly in the walls of blood vessels in the deep layers of the polymicrogyric cortex. There was no neuronal over-migration and the pial surface was intact. However, there were also areas of gliosis and centrilobular sclerosis in the cerebellum, in a pattern indicating ischaemic tissue damage and atrophy [74]. The co-existence of PMG and ischaemic atrophy may be explained by abnormal endothelial function leading to reduced perfusion or exchange at the neurovascular unit and manifesting as either malformation or atrophy according to specific vulnerability at different periods of development. Whether the malformation is the result of an independent and direct influence of occludin on neuronal migration has not, to our knowledge, been explored.

#### COL4A1/COL4A2

Type 4 collagen is ubiquitous in basement membranes and mutations in *COL4A1/COL4A2* are associated with porencephaly, schizencephaly and cobblestone cortex with normal dystroglycan [28,75]. Mutations in collagen genes may affect cortical development either by disrupting vascular membranes or the membranes covering the brain surface. Autopsy studies of babies with mutations in this gene have not been described to date.

#### ALX4 and MSX2

These genes are involved in control of membranous ossification but PMG is described in some patients with mutations in these genes. The typical constellation of findings includes parietal foramina, aplasia of the lateral end of the clavicle and malformations of the dural sinuses; a number of patients have been described with occipital PMG, in some there was associated white matter abnormality on imaging, encephalomalacia and strokes [76,77]. This was associated with dural venous malformation characterized by persistent median prosencephalic vein and an underdeveloped straight sinus. Aberrant venous drainage from the occipital lobes in early fetal life would explain PMG and, in later fetal and postnatal life, the encephalomalacia and strokes. However increasing recognition of the role of the meninges in cortical development [26,78] indicates that their direct involvement in PMG associated with *ALX4* and *MSX2* mutations cannot be dismissed.

#### 22Q11

PMG has been described pathologically in patients with deletions in *22Q11* and MRI has shown cerebral atrophy and loss of white matter volume [4,79]. It is likely that the abnormal embryonic vascular development in this condition is associated with hypoperfusion which leads to malformation, rather than any independent gene effect on cortical development [80].

#### Sturge Weber Syndrome (SWS)

Somatic activating mutations in *GNAQ* have been identified in affected tissue from SWS and port-wine stains [81]. Focal PMG has been reported in some patients with SWS, typically in the cortex ipsilateral to a leptomeningeal angioma. This has been assumed to be due to chronic hypoxia as a result of hypoperfusion in the cortex underlying the meningeal angioma. However, the cortical pathology beneath the angioma is not simply that of ischaemia and perfusion and metabolic abnormalities extend beyond the cortex beneath the angioma. The mechanisms of cortical damage remain unclear [82-84].

### PMG associated with genetic etiologies

To date mutations in a large number of genes have been associated with PMG [4]. However, none has yet been identified in which PMG is the phenotypic hallmark; it remains only one of a range of brain abnormalities including pachygyria, lissencephaly, schizencephaly, callosal, hippocampal, brainstem and cerebellar abnormalities. PMG can be associated with mutations in genes encoding for proteins with a wide variety of functions, including centrosome-, cilia- and microtubule-related proteins, or proteins involved in maintaining the integrity of the basement membrane [85,86]. Many of these studies depend on the description of the human cortical malformation by MRI. More precise definition of the pathology in the human brain, together with animal models, are beginning to identify the specific pathways disturbed in individual syndromes. The unfolding story of some of the more important genes related to PMG demonstrates the significance of the tubulins and GPR56. Many other genes have been associated with PMG, we note briefly those where the diagnosis has been verified by pathology.

#### Tubulins

Multiple tubulins are found in post mitotic cells of the human nervous system and are required to build microtubules which are essential for neuronal migration, differentiation and axonal guidance [87]. PMG has been reported in patients with mutations in *TUBA1A, TUBB2B, TUBB3 and TUBA8* [3,7,16,88]. Closer analysis of the pattern of PMG in some cases with tubulin mutations shows breaches in the pial basement membrane, over-migration of neurones, disorganisation of radial glial fibres and cerebellar nodular heterotopias [7].

#### GPR56

**Figure 13:**
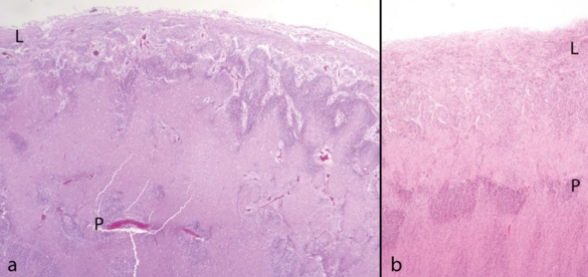
***GPR56*** **mutation and cobblestone cortex. a**: *GPR56* mutation in fetus of 27 weeks. There are irregular remnants of the original cortical surface (P) buried beneath a band of white matter. Above this the cortical ribbon is festooned beneath greatly thickened and vascular leptomeninges (L). **b**: Cobblestone malformation, fetus 20 weeks. There is a more obvious linear remnant of the cortical surface (P). Beneath it are clusters of neurones of the original cortical plate abutting the pial surface. Above is a smooth band of ectopic cortical cells mixed with vascular meninges over the brain surface (L). (**a** and **b** H&E).

Based on radiological studies, PMG associated with *GPR56* mutation was classified as localised bilateral frontoparietal PMG [89], but subsequent MRI and pathological studies show that in this condition PMG and cobblestone malformation co-exist [53,89]. Animal models [31] show focal defects in the pial basement membrane and over-migration of neurones into the leptomeninges; they have established a role for GPR56 in maintaining basement membrane integrity and adhesion of migrating cells to specific components of basement membrane [27]. The histology and distribution of the cortical pathology in human *GPR56* mutation show subtle differences from the cobblestone malformation associated with dystroglycanopathies (Figure 13). Thickened and metaplastic cerebellar leptomeninges containing skeletal muscle fibres have been described [see Jansen A, Robitaille Y, Honavar M, Mullatti N, Leventer RJ, Andermann E, Andermann F, Squier W (forthcoming) The histopathology of polymicrogyria: a series of 71 brain autopsy studies].

#### WDR62

Mutations in this gene are associated with microcephaly and multiple brain malformations [90]. WDR62 protein is expressed in neural progenitors in the neocortex transiently during the period of embryonic neurogenesis [85,91]. It is a centrosomal as well as a nuclear protein and the localization is dependent both on the cell phase and on the cell type [92]. Radiological studies have suggested that PMG with or without schizencephaly is seen in patients with *WDR62* mutation. The only one case with pathology, a 27 week human fetus, had a thin disorganised cortical plate, focal leptomeningeal over-migration and subcortical heterotopia but no PMG.

#### Monosomy of 1p36

Detailed neuropathological analysis of a single case revealed festooning of the cortex without fusion or surface abnormality. The authors debated whether to describe this malformation as PMG; it is consistent with our inclusive categorisation. Interestingly the malformation was restricted to dorsolateral aspects of the cerebral hemispheres and was associated with hippocampal malrotation, but not dysplasia, and periventricular heterotopia [17].

#### EOMES

PMG, microcephaly and agenesis of the corpus callosum are described in inactivation of the T-box transcription factor gene *EOMES* [52]. *EOMES* codes a transcription factor which influences regional gene expression and is involved in implementing regional identity in the cortex [51].

#### LAMC3

Laminins are extracellular adhesion proteins usually localised to basement membranes. LAMC3 also binds either to nidogen or to α6β1-integrin and recessive mutations in this gene are associated with occipital cortical malformation including PMG [93].

### PMG associated with metabolic disorders

PMG is described in a number of metabolic disorders including Zellweger’s syndrome [94], congenital disorders of glycosylation, as well as mitochondrial diseases such as Leigh’s disease and PDH deficiency [4,95]. Abnormal cytosomes in radial glia and migrating neurones have been implicated in the cortical malformation in Zellweger’s syndrome [96].

#### PMG associated with other brain malformations

PMG is associated with a number of other brain malformations [1]. Those which appear to contribute some insight into pathogenic mechanisms (cobblestone malformation, GPR56 mutation) are noted above. Others are beyond the scope of this review.

## Conclusions

Abnormality of the brain surface over polymicrogyric cortex is the commonest finding in PMG of any cause. This underlines the importance of the brain surface, particularly pial cells and basement membrane, in the control of normal corticogenesis. The physical and mechanical effects of thickened and collagenous leptomeninges potentially influence folding of the underlying developing cortex. It remains unclear however, to what extent the changes at the brain surface contribute to the development of PMG or result from the same underlying cause.

Fusion of the adjacent meningeal surfaces of gyri is common and may leave traces identified by large blood vessels and collagen fibres communicating with the surface in areas of PMG. In only one circumstance have we encountered fusion of adjacent gyral surfaces in the absence of leptomeningeal surface abnormality and overmigration of cells into the leptomeninges.

Abnormal neuronal festooning of the neuronal layer beneath a smooth surface appears in some cases to be the primary malformation, occurring well before the onset of normal gyration. This supports the argument that PMG is not merely an abnormality of folding or fusion of adjacent gyri but in some cases reflects an earlier inherent anomaly of cortical development.

Many questions remain unanswered and it is hoped that the integration of imaging, genetics and animal models with human observational neuropathology will assist in finding at least some of the answers. To date, it has not been possible to determine the primary insult, genetic or destructive, associated with each of the observed subtypes of PMG.

## Additional file

Additional file 1:
**Mechanisms of Polymicrogyria.**

## References

[1] Barkovich AJ (2010). Current concepts of polymicrogyria. Neuroradiology.

[2] Guerrini R, Dobyns WB, Barkovich AJ (2008). Abnormal development of the human cerebral cortex: genetics, functional consequences and treatment options. Trends Neurosci.

[3] Leventer RJ, Jansen A, Pilz DT, Stoodley N, Marini C, Dubeau F, Malone J, Mitchell LA, Mandelstam S, Scheffer IE, Berkovic SF, Andermann F, Andermann E, Guerrini R, Dobyns WB (2010). Clinical and imaging heterogeneity of polymicrogyria: a study of 328 patients. Brain.

[4] Jansen A, Andermann E (2005). Genetics of the polymicrogyria syndromes. J Med Genet.

[5] Villard L, Nguyen K, Cardoso C, Martin CL, Weiss AM, Sifry-Platt M, Grix AW, Graham JM, Winter RM, Leventer RJ, Dobyns WB (2002). A locus for bilateral perisylvian polymicrogyria maps to Xq28. Am J Hum Genet.

[6] Santos NF, Secolin R, Brandao-Almeida IL, Silva MS, Torres FR, Tsuneda SS, Guimaraes CA, Hage SR, Cendes F, Guerreiro MM, Lopes-Cendes I (2008). A new candidate locus for bilateral perisylvian polymicrogyria mapped on chromosome Xq27. Am J Med Genet A.

[7] Jaglin XH, Poirier K, Saillour Y, Buhler E, Tian G, Bahi-Buisson N, Fallet-Bianco C, Phan-Dinh-Tuy F, Kong XP, Bomont P, Castelnau-Ptakhine L, Odent S, Loget P, Kossorotoff M, Snoeck I, Plessis G, Parent P, Beldjord C, Cardoso C, Represa A, Flint J, Keays DA, Cowan NJ, Chelly J (2009). Mutations in the beta-tubulin gene TUBB2B result in asymmetrical polymicrogyria. Nat Genet.

[8] Barth PG (1984). Prenatal clastic encephalopathies. Clin Neurol Neurosurg.

[9] Dekaban A (1965). Large defects in cerebral hemispheres associated with cortical dysgenesis. J Neuropathol Exp Neurol.

[10] Richman DP, Stewart RM, Caviness VS (1974). Cerebral microgyria in a 27-week fetus: an architectonic and topographic analysis. J Neuropathol Exp Neurol.

[11] Squier MV (1993). Development of the cortical dysplasia of type II lissencephaly. Neuropathol Appl Neurobiol.

[12] Friede RL, Friede RL (1989). Dysplasias of the Cerebral Cortex. Developmental Neuropathology.

[13] Norman MG, Becker LE, Cochrane DD, Muenke M (1995). Congenital Malformations of the Brain: Pathologic, Embryologic, Clinical, Radiological and Genetic Aspects.

[14] Judkins AR, Martinez D, Ferreira P, Dobyns WB, Golden JA (2011). Polymicrogyria includes fusion of the molecular layer and decreased neuronal populations but normal cortical laminar organization. J Neuropathol Exp Neurol.

[15] Barth PG (1987). Disorders of neuronal migration. Can J Neurol Sci.

[16] Fallet-Bianco C, Loeuillet L, Poirier K, Loget P, Chapon F, Pasquier L, Saillour Y, Beldjord C, Chelly J, Francis F (2008). Neuropathological phenotype of a distinct form of lissencephaly associated with mutations in TUBA1A. Brain.

[17] Shiba N, Daza RA, Shaffer LG, Barkovich AJ, Dobyns WB, Hevner RF (2013). Neuropathology of brain and spinal malformations in a case of monosomy 1p36. Acta Neuropathol Commun.

[18] Siegenthaler JA, Pleasure SJ (2011). We have got you ‘covered’: how the meninges control brain development. Curr Opin Genet Dev.

[19] Dvorak K, Feit J, Jurankova Z (1978). Experimentally induced focal microgyria and status verrucosus deformis in rats–pathogenesis and interrelation: histological and autoradiographical study. Acta Neuropathol.

[20] Ferrer I, Alcantara S, Catala I, Zujar MJ (1993). Experimentally induced laminar necrosis, status verrucosus, focal cortical dysplasia reminiscent of microgyria, and porencephaly in the rat. Exp Brain Res.

[21] Radakovits R, Barros CS, Belvindrah R, Patton B, Muller U (2009). Regulation of radial glial survival by signals from the meninges. J Neurosci.

[22] Barth PG, van der Harten JJ (1985). Parabiotic twin syndrome with topical isocortical disruption and gastroschisis. Acta Neuropathol.

[23] Devisme L, Bouchet C, Gonzales M, Alanio E, Bazin A, Bessieres B, Bigi N, Blanchet P, Bonneau D, Bonnieres M, Bucourt M, Carles D, Clarisse B, Delahaye S, Fallet-Bianco C, Figarella-Branger D, Gaillard D, Gasser B, Delezoide AL, Guimiot F, Joubert M, Laurent N, Laquerriere A, Liprandi A, Loget P, Marcorelles P, Martinovic J, Menez F, Patrier S, Pelluard F (2012). Cobblestone lissencephaly: neuropathological subtypes and correlations with genes of dystroglycanopathies. Brain J Neurol.

[24] Towfighi J, Sassani JW, Suzuki K, Ladda RL (1984). Cerebro-ocular dysplasia-muscular dystrophy (COD-MD) syndrome. Acta Neuropathol.

[25] Streeter G (1915). The developmant of the venous sinuses of the dura mater in the human embryo. Am J Anat.

[26] Halfter W, Dong S, Yip YP, Willem M, Mayer U (2002). A critical function of the pial basement membrane in cortical histogenesis. J Neurosci.

[27] Koirala S, Jin Z, Piao X, Corfas G (2009). GPR56-regulated granule cell adhesion is essential for rostral cerebellar development. J Neurosci.

[28] Labelle-Dumais C, Dilworth DJ, Harrington EP, de Leau M, Lyons D, Kabaeva Z, Manzini MC, Dobyns WB, Walsh CA, Michele DE, Gould DB (2011). COL4A1 mutations cause ocular dysgenesis, neuronal localization defects, and myopathy in mice and Walker-Warburg syndrome in humans. PLoS Genet.

[29] Hu H, Yang Y, Eade A, Xiong Y, Qi Y (2007). Breaches of the pial basement membrane and disappearance of the glia limitans during development underlie the cortical lamination defect in the mouse model of muscle-eye-brain disease. J Comp Neurol.

[30] Blackshear PJ, Silver J, Nairn AC, Sulik KK, Squier MV, Stumpo DJ, Tuttle JS (1997). Widespread neuronal ectopia associated with secondary defects in cerebrocortical chondroitin sulfate proteoglycans and basal lamina in MARCKS-deficient mice. Exp Neurol.

[31] Li S, Jin Z, Koirala S, Bu L, Xu L, Hynes RO, Walsh CA, Corfas G, Piao X (2008). GPR56 regulates pial basement membrane integrity and cortical lamination. J Neurosci.

[32] Sievers J, Pehlemann FW, Gude S, Berry M (1994). Meningeal cells organize the superficial glia limitans of the cerebellum and produce components of both the interstitial matrix and the basement membrane. J Neurocytol.

[33] Aldinger KA, Lehmann OJ, Hudgins L, Chizhikov VV, Bassuk AG, Ades LC, Krantz ID, Dobyns WB, Millen KJ (2009). FOXC1 is required for normal cerebellar development and is a major contributor to chromosome 6p25.3 Dandy-Walker malformation. Nat Genet.

[34] Siegenthaler JA, Ashique AM, Zarbalis K, Patterson KP, Hecht JH, Kane MA, Folias AE, Choe Y, May SR, Kume T, Napoli JL, Peterson AS, Pleasure SJ (2009). Retinoic acid from the meninges regulates cortical neuron generation. Cell.

[35] Choe Y, Siegenthaler JA, Pleasure SJ (2012). A cascade of morphogenic signaling initiated by the meninges controls corpus callosum formation. Neuron.

[36] Zarbalis K, Choe Y, Siegenthaler JA, Orosco LA, Pleasure SJ (2012). Meningeal defects alter the tangential migration of cortical interneurons in Foxc1hith/hith mice. Neural Dev.

[37] Lopez-Bendito G, Sanchez-Alcaniz JA, Pla R, Borrell V, Pico E, Valdeolmillos M, Marin O (2008). Chemokine signaling controls intracortical migration and final distribution of GABAergic interneurons. J Neurosci.

[38] Frotscher M (1998). Cajal-Retzius cells, Reelin, and the formation of layers. Curr Opin Neurobiol.

[39] Eriksson SH, Thom M, Heffernan J, Lin WR, Harding BN, Squier MV, Sisodiya SM (2001). Persistent reelin-expressing Cajal-Retzius cells in polymicrogyria. Brain.

[40] Meyer G (2010). Building a human cortex: the evolutionary differentiation of Cajal-Retzius cells and the cortical hem. J Anat.

[41] Borrell V, Marin O (2006). Meninges control tangential migration of hem-derived Cajal-Retzius cells via CXCL12/CXCR4 signaling. Nat Neurosci.

[42] Rakic P (2007). The radial edifice of cortical architecture: from neuronal silhouettes to genetic engineering. Brain Res Rev.

[43] Guerrini R, Parrini E (2010). Neuronal migration disorders. Neurobiol Dis.

[44] Wieck G, Leventer RJ, Squier WM, Jansen A, Andermann E, Dubeau F, Ramazzotti A, Guerrini R, Dobyns WB (2005). Periventricular nodular heterotopia with overlying polymicrogyria. Brain.

[45] Friede RL (1989). Devlopmental Neuropathology.

[46] Leyten QH, Renkawek K, Renier WO, Gabreels FJ, Mooy CM, ter Laak HJ, Mullaart RA (1991). Neuropathological findings in muscle-eye-brain disease (MEB-D): neuropathological delineation of MEB-D from congenital muscular dystrophy of the Fukuyama type. Acta Neuropathol.

[47] Gelot A, Billette de Villemeur T, Bordarier C, Ruchoux MM, Moraine C, Ponsot G (1995). Developmental aspects of type II lissencephaly: comparative study of dysplastic lesions in fetal and post-natal brains. Acta Neuropathol.

[48] Squier W, Jansen A (2010). Abnormal development of the human cerebral cortex. J Anat.

[49] Jissendi-Tchofo P, Kara S, Barkovich AJ (2009). Midbrain-hindbrain involvement in lissencephalies. Neurology.

[50] Ten Donkelaar HJ, Lammens M, Hori A (2006). Clinical Neuro-embryology.

[51] Elsen GE, Hodge RD, Bedogni F, Daza RA, Nelson BR, Shiba N, Reiner SL, Hevner RF (2013). The protomap is propagated to cortical plate neurons through an Eomes-dependent intermediate map. Proc Natl Acad Sci U S A.

[52] Baala L, Briault S, Etchevers HC, Laumonnier F, Natiq A, Amiel J, Boddaert N, Picard C, Sbiti A, Asermouh A, Attie-Bitach T, Encha-Razavi F, Munnich A, Sefiani A, Lyonnet S (2007). Homozygous silencing of T-box transcription factor EOMES leads to microcephaly with polymicrogyria and corpus callosum agenesis. Nat Genet.

[53] Bahi-Buisson N, Poirier K, Boddaert N, Fallet-Bianco C, Specchio N, Bertini E, Caglayan O, Lascelles K, Elie C, Rambaud J, Baulac M, An I, Dias P, des Portes V, Moutard ML, Soufflet C, El Maleh M, Beldjord C, Villard L, Chelly J (2010). GPR56-related bilateral frontoparietal polymicrogyria: further evidence for an overlap with the cobblestone complex. Brain J Neurol.

[54] Weimer JM, Yokota Y, Stanco A, Stumpo DJ, Blackshear PJ, Anton ES (2009). MARCKS modulates radial progenitor placement, proliferation and organization in the developing cerebral cortex. Development.

[55] Clement E, Mercuri E, Godfrey C, Smith J, Robb S, Kinali M, Straub V, Bushby K, Manzur A, Talim B, Cowan F, Quinlivan R, Klein A, Longman C, McWilliam R, Topaloglu H, Mein R, Abbs S, North K, Barkovich AJ, Rutherford M, Muntoni F (2008). Brain involvement in muscular dystrophies with defective dystroglycan glycosylation. Ann Neurol.

[56] Jiang X, Iseki S, Maxson RE, Sucov HM, Morriss-Kay GM (2002). Tissue origins and interactions in the mammalian skull vault. Dev Biol.

[57] Altman J, Bayer SA (1978). Prenatal development of the cerebellar system in the rat. II. Cytogenesis and histogenesis of the inferior olive, pontine gray, and the precerebellar reticular nuclei. J Comp Neurol.

[58] Altman J, Bayer SA (1978). Prenatal development of the cerebellar system in the rat. I. Cytogenesis and histogenesis of the deep nuclei and the cortex of the cerebellum. J Comp Neurol.

[59] Gaytant MA, Rours GI, Steegers EA, Galama JM, Semmekrot BA (2003). Congenital cytomegalovirus infection after recurrent infection: case reports and review of the literature. Eur J Pediatr.

[60] Teissier N, Fallet-Bianco C, Delezoide AL, Laquerriere A, Marcorelles P, Khung-Savatovsky S, Nardelli J, Cipriani S, Csaba Z, Picone O, Golden JA, Van Den Abbeele T, Gressens P, Adle-Biassette H (2014). Cytomegalovirus-induced brain malformations in fetuses. J Neuropathol Exp Neurol.

[61] Kenneson A, Cannon MJ (2007). Review and meta-analysis of the epidemiology of congenital cytomegalovirus (CMV) infection. Rev Med Virol.

[62] Span AH, Frederik PM, Grauls G, Van Boven GP, Bruggeman CA (1993). CMV induced vascular injury: an electron-microscopic study in the rat. In Vivo.

[63] Tsutsui Y, Kosugi I, Kawasaki H (2005). Neuropathogenesis in cytomegalovirus infection: indication of the mechanisms using mouse models. Rev Med Virol.

[64] Norman RM, Urich H, Woods GE (1958). The relationship between prenatal porencephaly and the encephalomalacias of early life. J Ment Sci.

[65] Ferrer I, Catala I (1991). Unlayered polymicrogyria: structural and developmental aspects. Anat Embryol (Berl).

[66] Guerrini R, Dubeau F, Dulac O, Barkovich AJ, Kuzniecky R, Fett C, Jones-Gotman M, Canapicchi R, Cross H, Fish D, Bonanni P, Jambaque I, Andermann F (1997). Bilateral parasagittal parietooccipital polymicrogyria and epilepsy. Ann Neurol.

[67] Huang BY, Castillo M (2008). Hypoxic-ischemic brain injury: imaging findings from birth to adulthood. Radiographics.

[68] Williams RS, Ferrante RJ, Caviness VS (1976). The cellular pathology of microgyria: a Golgi analysis. Acta Neuropathol.

[69] Paolicelli RC, Bolasco G, Pagani F, Maggi L, Scianni M, Panzanelli P, Giustetto M, Ferreira TA, Guiducci E, Dumas L, Ragozzino D, Gross CT (2011). Synaptic pruning by microglia is necessary for normal brain development. Science.

[70] Nitschke Y, Baujat G, Botschen U, Wittkampf T, du Moulin M, Stella J, Le Merrer M, Guest G, Lambot K, Tazarourte-Pinturier MF, Chassaing N, Roche O, Feenstra I, Loechner K, Deshpande C, Garber SJ, Chikarmane R, Steinmann B, Shahinyan T, Martorell L, Davies J, Smith WE, Kahler SG, McCulloch M, Wraige E, Loidi L, Hohne W, Martin L, Hadj-Rabia S, Terkeltaub R (2012). Generalized arterial calcification of infancy and pseudoxanthoma elasticum can be caused by mutations in either ENPP1 or ABCC6. Am J Hum Genet.

[71] Ramjan KA, Roscioli T, Rutsch F, Sillence D, Munns CF (2009). Generalized arterial calcification of infancy: treatment with bisphosphonates. Nat Clin Pract Endocrinol Metab.

[72] Yakovlev PI, Wadsworth RC (1946). Schizencephalies: a study of the congenital clefts in the cerebral mantle. I. Clefts with fused lips. J Neuropathol Exp Neurol.

[73] Du D, Xu F, Yu L, Zhang C, Lu X, Yuan H, Huang Q, Zhang F, Bao H, Jia L, Wu X, Zhu X, Zhang X, Zhang Z, Chen Z (2010). The tight junction protein, occludin, regulates the directional migration of epithelial cells. Dev Cell.

[74] O’Driscoll MC, Daly SB, Urquhart JE, Black GC, Pilz DT, Brockmann K, McEntagart M, Abdel-Salam G, Zaki M, Wolf NI, Ladda RL, Sell S, D’Arrigo S, Squier W, Dobyns WB, Livingston JH, Crow YJ (2010). Recessive mutations in the gene encoding the tight junction protein occludin cause band-like calcification with simplified gyration and polymicrogyria. Am J Hum Genet.

[75] Yoneda Y, Haginoya K, Arai H, Yamaoka S, Tsurusaki Y, Doi H, Miyake N, Yokochi K, Osaka H, Kato M, Matsumoto N, Saitsu H (2012). De novo and inherited mutations in COL4A2, encoding the type IV collagen alpha2 chain cause porencephaly. Am J Hum Genet.

[76] Reddy AT, Hedlund GL, Percy AK (2000). Enlarged parietal foramina: association with cerebral venous and cortical anomalies. Neurology.

[77] Valente KD, Valente M (2004). Epilepsy in one family with parietal foramina: an incidental finding?. J Neurol Neurosurg Psychiatry.

[78] Zarbalis K, Siegenthaler JA, Choe Y, May SR, Peterson AS, Pleasure SJ (2007). Cortical dysplasia and skull defects in mice with a Foxc1 allele reveal the role of meningeal differentiation in regulating cortical development. Proc Natl Acad Sci USA.

[79] Bingham PM, Lynch D, McDonald-McGinn D, Zackai E (1998). Polymicrogyria in chromosome 22 delection syndrome. Neurology.

[80] Robin NH, Taylor CJ, McDonald-McGinn DM, Zackai EH, Bingham P, Collins KJ, Earl D, Gill D, Granata T, Guerrini R, Katz N, Kimonis V, Lin JP, Lynch DR, Mohammed SN, Massey RF, McDonald M, Rogers RC, Splitt M, Stevens CA, Tischkowitz MD, Stoodley N, Leventer RJ, Pilz DT, Dobyns WB (2006). Polymicrogyria and deletion 22q11.2 syndrome: window to the etiology of a common cortical malformation. Am J Med Genet A.

[81] Shirley MD, Tang H, Gallione CJ, Baugher JD, Frelin LP, Cohen B, North PE, Marchuk DA, Comi AM, Pevsner J (2013). Sturge-Weber syndrome and port-wine stains caused by somatic mutation in GNAQ. N Engl J Med.

[82] Comi AM (2003). Pathophysiology of Sturge-Weber syndrome. J Child Neurol.

[83] Alkonyi B, Miao Y, Wu J, Cai Z, Hu J, Chugani HT, Juhasz C (2012). A perfusion-metabolic mismatch in Sturge-Weber syndrome: a multimodality imaging study. Brain Dev.

[84] McCartney E, Squier W (2014) Patterns and pathways of calcification in the developing brain. Developmental Medicine and Child Neurology online May 2014 doi:10.1111/dmcn.12493 10.1111/dmcn.12493 24844884

[85] Bilguvar K, Ozturk AK, Louvi A, Kwan KY, Choi M, Tatli B, Yalnizoglu D, Tuysuz B, Caglayan AO, Gokben S, Kaymakcalan H, Barak T, Bakircioglu M, Yasuno K, Ho W, Sanders S, Zhu Y, Yilmaz S, Dincer A, Johnson MH, Bronen RA, Kocer N, Per H, Mane S, Pamir MN, Yalcinkaya C, Kumandas S, Topcu M, Ozmen M, Sestan N (2010). Whole-exome sequencing identifies recessive WDR62 mutations in severe brain malformations. Nature.

[86] Poirier K, Lebrun N, Broix L, Tian G, Saillour Y, Boscheron C, Parrini E, Valence S, Pierre BS, Oger M, Lacombe D, Genevieve D, Fontana E, Darra F, Cances C, Barth M, Bonneau D, Bernadina BD, N’Guyen S, Gitiaux C, Parent P, Des Portes V, Pedespan JM, Legrez V, Castelnau-Ptakine L, Nitschke P, Hieu T, Masson C, Zelenika D, Andrieux A (2013). Mutations in TUBG1, DYNC1H1, KIF5C and KIF2A cause malformations of cortical development and microcephaly. Nat Genet.

[87] Tischfield MA, Cederquist GY, Gupta ML, Engle EC (2011). Phenotypic spectrum of the tubulin-related disorders and functional implications of disease-causing mutations. Curr Opin Genet Dev.

[88] Poirier K, Saillour Y, Bahi-Buisson N, Jaglin XH, Fallet-Bianco C, Nabbout R, Castelnau-Ptakhine L, Roubertie A, Attie-Bitach T, Desguerre I, Genevieve D, Barnerias C, Keren B, Lebrun N, Boddaert N, Encha-Razavi F, Chelly J (2010). Mutations in the neuronal ss-tubulin subunit TUBB3 result in malformation of cortical development and neuronal migration defects. Hum Mol Genet.

[89] Piao X, Hill RS, Bodell A, Chang BS, Basel-Vanagaite L, Straussberg R, Dobyns WB, Qasrawi B, Winter RM, Innes AM, Voit T, Ross ME, Michaud JL, Descarie JC, Barkovich AJ, Walsh CA (2004). G protein-coupled receptor-dependent development of human frontal cortex. Science.

[90] Manzini MC, Walsh CA (2011). What disorders of cortical development tell us about the cortex: one plus one does not always make two. Curr Opin Genet Dev.

[91] Yu TW, Mochida GH, Tischfield DJ, Sgaier SK, Flores-Sarnat L, Sergi CM, Topcu M, McDonald MT, Barry BJ, Felie JM, Sunu C, Dobyns WB, Folkerth RD, Barkovich AJ, Walsh CA (2010). Mutations in WDR62, encoding a centrosome-associated protein, cause microcephaly with simplified gyri and abnormal cortical architecture. Nat Genet.

[92] Bhat V, Girimaji SC, Mohan G, Arvinda HR, Singhmar P, Duvvari MR, Kumar A (2011). Mutations in WDR62, encoding a centrosomal and nuclear protein, in Indian primary microcephaly families with cortical malformations. Clin Genet.

[93] Barak T, Kwan KY, Louvi A, Demirbilek V, Saygi S, Tuysuz B, Choi M, Boyaci H, Doerschner K, Zhu Y, Kaymakcalan H, Yilmaz S, Bakircioglu M, Caglayan AO, Ozturk AK, Yasuno K, Brunken WJ, Atalar E, Yalcinkaya C, Dincer A, Bronen RA, Mane S, Ozcelik T, Lifton RP, Sestan N, Bilguvar K, Gunel M (2011). Recessive LAMC3 mutations cause malformations of occipital cortical development. Nat Genet.

[94] Zellweger H (1987). The cerebro-hepato-renal (Zellweger) syndrome and other peroxisomal disorders. Dev Med Child Neurol.

[95] van Straaten HL, van Tintelen JP, Trijbels JM, van den Heuvel LP, Troost D, Rozemuller JM, Duran M, de Vries LS, Schuelke M, Barth PG (2005). Neonatal lactic acidosis, complex I/IV deficiency, and fetal cerebral disruption. Neuropediatrics.

[96] Powers JM (2001). Normal and defective neuronal membranes: structure and function: neuronal lesions in peroxisomal disorders. J Mol Neurosci.

