# Supplementary material for: Polymicrogyria: pathology, fetal origins and mechanisms
Source: Acta Neuropathol Commun. 2014 Jul 22;2:80. doi: 10.1186/s40478-014-0080-3 (PMC4149230; doi:10.1186/s40478-014-0080-3)
Supplement: Additional file 1: — Mechanisms of Polymicrogyria. [file 40478_2014_80_MOESM1_ESM.docx]

**Mechanisms of Polymicrogyria**

| ***Disorders of the brain surface***  Most PMG is associated with pathology at the brain surface. This is a complex unit comprising leptomeningeal cells, basement membrane, intrinsic cortical cells and glial end feet anchoring the radial guide cells. Leptomeningeal cells secrete the basement membrane and produce signalling factors responsible for corticogenesis.  Physical breaks in the basement membrane lead to overmigration while altered chemical properties may lead to fusion of adjacent surfaces.  Loss of integrity of the surface/radial glial unit occurs when there is tissue destruction due to infections, ischaemia and metabolic disorders. |
| --- |
| ***Abnormal surface fusion***  Any abnormality of the brain surface may result in alteration of its physical properties including increased stickiness allowing adhesion of adjacent gyral surfaces and the contiguous medial frontal lobes. |
| ***Premature cortical folding***  Rarely PMG results from abnormal cortical folding identified well before normal cortical gyration is expected to occur. |
| ***Temporospatial patterning of PMG***  Analysis of the temporospatial patterning of PMG and other cortical malformations supports the view that incomplete migration or loss of signalling functions of leptomeningeal may be significant factors in the aetiology of PMG. |
| ***Physical and Mechanical Constraints***  Surface abnormalities with increased collagen deposition are common in PMG. The altered mechanical properties and increased stiffness of the brain surface may constrain the developing neuronal band leading to increased packing density of its folds and PMG |
